# Genetic Variation in the β2-Adrenocepter Gene Is Associated with Susceptibility to Bacterial Meningitis in Adults

**DOI:** 10.1371/journal.pone.0037618

**Published:** 2012-05-18

**Authors:** Kirsten S. Adriani, Matthijs C. Brouwer, Frank Baas, Aeilko H. Zwinderman, Arie van der Ende, Diederik van de Beek

**Affiliations:** 1 Department of Neurology, Center of Infection and Immunity Amsterdam (CINIMA), Academic Medical Center, University of Amsterdam, Amsterdam, The Netherlands; 2 Department of Genome Analysis, Center of Infection and Immunity Amsterdam (CINIMA), Academic Medical Center, University of Amsterdam, Amsterdam, The Netherlands; 3 Department of Biostatistics, Center of Infection and Immunity Amsterdam (CINIMA), Academic Medical Center, University of Amsterdam, Amsterdam, The Netherlands; 4 Department of Medical Microbiology, Center of Infection and Immunity Amsterdam (CINIMA), Academic Medical Center, University of Amsterdam, Amsterdam, The Netherlands; 5 Netherlands Reference Laboratory for Bacterial Meningitis, Center of Infection and Immunity Amsterdam (CINIMA), Academic Medical Center, University of Amsterdam, Amsterdam, The Netherlands; Oxford University, Viet Nam

## Abstract

Recently, the biased β2-adrenoceptor/β-arrestin pathway was shown to play a pivotal role in crossing of the blood brain barrier by *Neisseria meningitidis*. We hypothesized that genetic variation in the β2-adrenoceptor gene (*ADRB2*) may influence susceptibility to bacterial meningitis. In a prospective genetic association study we genotyped 542 patients with CSF culture proven community acquired bacterial meningitis and 376 matched controls for 2 functional single nucleotide polymorphisms in the β2-adrenoceptor gene (*ADRB2*). Furthermore, we analyzed if the use of non-selective beta-blockers, which bind to the β2-adrenoceptor, influenced the risk of bacterial meningitis. We identified a functional polymorphism in *ADRB2* (rs1042714) to be associated with an increased risk for bacterial meningitis (Odds ratio [OR] 1.35, 95% confidence interval [CI] 1.04–1.76; p = 0.026). The association remained significant after correction for age and was more prominent in patients with pneumococcal meningitis (OR 1.52, 95% CI 1.12–2.07; p = 0.007). For meningococcal meningitis the difference in genotype frequencies between patients and controls was similar to that in pneumococcal meningitis, but this was not statistically significant (OR 1.43, 95% CI 0.60–3.38; p = 0.72). Patients with bacterial meningitis had a lower frequency of non-selective beta-blockers use compared to the age matched population (0.9% *vs.* 1.8%), although this did not reach statistical significance (OR 1.96 [95% CI 0.88–4.39]; p = 0.09). In conclusion, we identified an association between a genetic variant in the β2-adrenoceptor and increased susceptibility to bacterial meningitis. The potential benefit of pharmacological treatment targeting the β2-adrenoceptor to prevent bacterial meningitis in the general population or patients with bacteraemia should be further studied in both experimental studies and observational cohorts.

## Introduction

Community-acquired bacterial meningitis is a disease with high mortality and morbidity, despite effective antimicrobial agents, adjunctive dexamethasone and implementation of childhood vaccination programmes [1]–[3]. *Streptococcus pneumoniae* and *Neisseria meningitidis* are the leading causes of bacterial meningitis in adults, with the first responsible for two thirds of cases in Europe and the United States [1], [4]. The disease is preceded by nasopharyngeal colonization, which occurs in up to 100% of the normal population for pneumococci and 18% for meningococci [5], [6]. Following nasopharyngeal colonization some of the bacteria are able to invade the bloodstream, avoid host defences and reach the blood-brain barrier [7]. The mechanism by which the bacteria cross the blood-brain barrier is not completely understood, but the interaction between host cell receptors and the bacteria is thought to contribute to transcytosis into the subarachnoid space [8].

Recently, Coureuil *et al.* showed an important role of the biased β2-adrenoceptor/β-arrestin pathway in the pathophysiology of meningococcal meningitis *in vitro*. The authors showed that *N. meningitidis* is able to use the β2-adrenoceptor/β-arrestin signalling pathway in endothelial cells to cross the blood-brain barrier [9]. The meningococcus was found to hijack the β2-adrenoceptor and thereby stabilize its binding to the endothelium. Subsequently, activation of the β-arrestin signalling pathway causes delocalization of junctional proteins, resulting in gaps in the blood-brain barrier through which the meningococcus invades the subarachnoid space. Previously, β-arrestin-1 was shown to participate in receptor-mediated transcytosis of *S. pneumoniae* as well [10].

The human β2-adrenoceptor has several functional variants determined by two single nucleotide polymorphisms (SNPs), rs1042713 and rs1042714 in the *ADRB2* gene [11]. Although the genetic variant of the receptor displayed normal agonist binding and functional coupling in a functional study, a markedly altered degree of agonist-promoted down regulation of receptor expression was shown [11]. Both SNPs have been associated with several diseases, such as asthma [12], [13]. Familial aggregation and genetic association studies have suggested a genetic influence on susceptibility to pneumococcal and meningococcal infections [14]. SNPs have also been suggested to influence the phenotype of disease, *i.e.* the development of meningitis or sepsis [15]. We hypothesized that crossing of the blood-brain barrier by microorganisms such as *N. meningitidis* and *S. pneumoniae*, the two most common causative bacteria of meningitis, may be influenced by these *ADRB2* SNPs. We further analysed if the use of beta-blockers influenced the risk of acquiring bacterial meningitis, as non-selective beta-blockers may limit the availability of β2-adrenoceptors for bacteria to cross the blood-brain barrier.

## Methods

We performed a prospective nationwide genetic association study on the influence of *ADRB2* SNP rs1042713 and rs1042714 on susceptibility to bacterial meningitis. In this study we included bacterial meningitis patients older than 16 years of age with positive cerebrospinal fluid (CSF) cultures who were identified by The Netherlands Reference Laboratory for Bacterial Meningitis (NRLBM) from March 2006 to June 2010 [3]. The NRLBM receives bacterial isolates from approximately 85% of bacterial meningitis patients in the Netherlands and provided the names of the hospitals where patients with bacterial meningitis had been admitted 2–6 days previously. The treating physician was contacted, and written informed consent was obtained from all participating patients or their legally authorized representatives. Patients could also be included by physicians familiar with the study through a 24/7 telephone service. Patients with hospital-acquired bacterial meningitis and negative CSF cultures were excluded. Controls for exposure/susceptibility were patients' partners or their non-related proxies living in the same dwelling, as household members are exposed to similar bacteria [16]. Furthermore, this choice of controls guaranteed similar socio-economic background of patients and controls. Data on age, sex and ethnicity of patients and controls were collected. For patients, information on medication use on admission was recorded. Use of beta-blockers was categorized in selective and non-selective beta-blockers. We compared the use of selective (β1) beta-blockers and non-selective (β1 and β2) blockers of bacterial meningitis patients with that of the age corrected general population.

Blood from patients and controls for DNA extraction was collected in sodium/EDTA tubes. DNA was isolated with the Gentra Puregene isolation kit (Qiagen, Hilden, Germany) and quality control procedures were performed to determine the yield of isolation. The rs1042713 and rs1042714 SNP were genotyped using TaqMan SNP Genotyping Assays (Applied Biosystems, Foster City, California, USA) in a LightCycler480 (Roche, Basel, Switzerland) using the Fast Start Universal Probe Mix (Roche) for rs1042713 and TaqMan Genotyping Master Mix (Applied Biosystems) for rs1042714, by the Genetics Core Facility in the Academic Medical Center, Amsterdam, the Netherlands. Laboratory personnel were blinded to clinical information. The Mann-Whitney U test was used to identify differences in baseline characteristics between groups with respect to continuous variables, and dichotomous variables were compared with use of the χ^2^ test. These statistical tests were 2-tailed, and a p-value of <0.05 was regarded as significant. Differences in genotype frequencies were analyzed with the χ^2^ or Fishers' exact tests by use of PASW18. Logistic regression analysis was used to analyse the difference in genotype frequency between patient and controls corrected for age. Subgroup analyses were performed for pneumococcal and for meningococcal meningitis patients. Furthermore, we separately compared the genotype frequency of bacterial meningitis patients with and without otitis media and/or sinusitis to controls. Since the *ADRB2* SNPs are thought to influence the blood-brain barrier crossing of bacteria, a potential effect is expected to be absent in patients with meningitis due to continuous infection from otitis media and/or sinusitis.

We calculated whether the genotype frequencies in white controls concurred with the Hardy Weinberg equilibrium (HWE) by use of a χ^2^ test with one degree of freedom with a p<0.001 to indicate significance. The study was approved by the research ethics committee of the Academic Medical Centre, Amsterdam, The Netherlands.

## Results

From March 2006 to June 2010, 734 patients with culture proven bacterial meningitis were included: in 534 (73%) cases *S. pneumoniae* was the causative organism, in 91 (12%) *N. meningitidis* and 109 (15%) were due to other microorganisms. DNA was available for 542 (74%) patients and 376 controls. The mean age of included patients was 55 years and 49.6% of the patients were male. Sex, age, ethnicity and socio-economic background were similar between patients and controls (Table 1). Clinical characteristics of the patient population are presented in Table 2.

**Table 1:** Age, sex and ethnicity of 542 patients and 376 controls.

Characteristic	Patients	Controls
Age	54.7 (±17)	55.7 (±15)
Male sex	269 (49.6%)	187 (49.7%)
Ethnicity		
White	510 (94%)	361 (96%)
African	24 (4.4%)	13 (3.5)
Asian	8 (1.5)	2 (0.5%)

**Table 2:** Clinical characteristics 542 patients with bacterial meningitis on admission and outcome.

Characteristics	No./no. of patients^a^
Mean age, yr (range)	55 (17–93)
Male/female sex – no.	269/273
Duration of symptoms <24 hours	233/520 (45)
Predisposing conditions	
Otitis media/sinusitis	192/542 (35)
Pneumonia	49/535 (9)
Immunocompromised state^b^	121/530 (23)
Symptoms on presentation	
Headache	413/535 (77)
Nausea	290/535 (54)
Neck stiffness	400/535 (75)
Temperature ≥38°C	408/535 (76)
Signs of septic shock^c^	270/525 (51)
Score on Glascow Coma Scale^d^	11 (9–14)
Altered mental status (Glascow Coma Scale <14)	385/531 (73)
Coma (Glascow Coma Scale <8 )	69/531 (13)
Outcome	
Unfavourable^f^	126/514 (23)
Death	36/514 (7)

a Data are number/number evaluated (%), and median (interquartile range) unless otherwise stated.

b Immunocompromised was defined as the use of immunosuppressive drugs, the presence of diabetes mellitus or alcoholism, and patients with the human immunodeficiency virus (HIV).

c Defined as a systolic blood pressure ≤90 mmHg, a diastolic blood pressure <60 mmHg and/or heart rate ≥120/min.

d Unfavourable outcome was defined as a Glascow Outcome Score (GOS) <5, a score of 1 indicates death, 2 indicates vegetative state, 3 indicates severe disability, 4 indicates moderate disability and 5 indicates mild or no disability.

Genotyping was successful in 99.3% of the samples and the genotype frequency of controls for both SNPs concurred with the Hardy-Weinberg Equilibrium. Genotype frequencies were similar between patients of different ethnic background. The Gln/Glu genotype of the rs1042714 was associated with increased susceptibility to bacterial meningitis (Table 3). The genotype was found in 271 of 542 (50%) patients and 160 of 376 (42%) controls (odds ratio [OR] 1.35, 95% confidence interval [CI] 1.04–1.76; p = 0.026). After correction for age, rs1042714 was still significantly associated with susceptibility (OR 1.37, 95% CI 1.05–1.79; p = 0.023). The results were similar when the analysis was limited to white patients and controls (OR 1.36, 95% CI 1.04–1.79; p = 0.033). The genotype distribution of rs1042713 was similar between patients and controls. Subgroup analysis showed that the Gln/Glu genotype was found in 200 of 396 (51%) pneumococcal meningitis patients compared to 116 of 289 (40%) controls (OR 1.52, 95% CI 1.12–2.07; p = 0.007). For meningococcal meningitis the risk genotype showed a similar distribution. It was found in 36 of 69 (52%) patients and 13 of 30 (43%) controls, but this did not reach statistical significance due to the low numbers of patients (OR 1.43, 95% CI 0.60–3.38; p = 0.72). In the subgroup analysis of patients with otitis media and/or sinusitis, there was no association of rs1072714 and susceptibility (p = 0.15), while in patients without otitis media and/or sinusitis the rs1072714 genotype was still associated with increased susceptibility (OR 1.38, 95% CI 1.03–1.85; p = 0.030). Our study, however, lacked power to meet the standard of demonstrating a statistically significant interaction of otitis media and sinusitis by genotype. Both *ADRB2* SNPs were not associated the rate of unfavourable outcome or mortality rate.

**Table 3:** Genotype frequencies rs1042713 (R16G) and rs1042714 (Q27E) in bacterial meningitis patients and controls.

	Allele/genotype patients					Allele/genotype controls					Risk	p-value	Odds ratio
rs1042713	R	G	R/R	R/G	G/G	R	G	R/R	R/G	G/G	genotype		95% (CI^a^)
All patients	666	408	207	252	78	475	275	158	159	58	R/G	0.176	1.20 (0.92–1.57)
*S. pneumoniae*	489	297	151	187	55	446	306	143	160	73	R/G	0.087	1.31 (0.96–1.78)
*N. meningitidis*	87	49	29	29	10	46	14	17	12	1	G/G	0.100	5.00 (0.61–42.0)

rs1042714	Q	E	Q/Q	Q/E	E/E	Q	E	Q/Q	Q/E	E/E			
All patients	625	459	177	271	94	446	306	143	160	73	Q/E	0.026	1.35 (1.04–1.76)
*S. pneumoniae*	452	340	126	200	70	348	230	116	116	57	Q/E	0.007	1.52 (1.12–2.07)
*N. meningitidis*	60	78	12	36	21	29	31	8	13	9	Q/E	0.419	1.43 (0.60–3.38)

a Confidence interval.

Non-selective beta-blockers may present a means of preventing bacterial meningitis by blocking the β2-adrenoceptor, and are used by a substantial part of the population for hypertension and chronic heart failure [17]. A total of 104 of 642 patients (16%) for whom medication was specified used beta-blockers, of which 6 (0.9%) used non-selective beta-blockers and 98 (15%) β1-selective beta-blockers (Table 4). Predisposing factors for bacterial meningitis were present in all 6 patients using non-selective beta-blockers, 56 (57%) patients using selective beta-blockers and 278 (52%) of patients using no beta-blockers (p = 0.041). Four of the patients on non-selective beta-blockers presented with otitis media and four had a history of diabetes mellitus and were therefore immunocompromised. Data on beta-blocker use in the control population was not available to assess if non-selective beta-blocker use decreased the risk of bacterial meningitis. Data from the national pharmaceutical registry (Foundation of Pharmaceutical Statistics) showed 12.9% of the age corrected general population use β1-selective beta-blockers and 1.8% non-selective beta-blockers [17]. A statistical trend toward lower use of non-selective beta-blockers and higher use of selective beta-blockers was observed in the meningitis cohort compared to the general population corrected for age (non-selective beta-blockers 0.9 *vs.* 1.8%; OR 1.96 [95% CI 0.88–4.39]; p = 0.09; selective beta-blockers 15.2% *vs.* 12.9%, OR 0.83 [95% CI 0.67–1.02], p = 0.08) [18].

**Table 4:** Use of beta-blockers in study bacterial meningitis patients, the general Dutch population and age corrected general population.

Characteristic	PatientsN = 638	General population^a^N = 16.575.000	Age corrected population^b^
Beta-blockers	103 (16%)	1.445.000 (8.7%)	2.480.000 (15.0%)
Non-selective	6 (0.8%)	191.000 (1.2%)	304.000 (1.8%)
Selective	97 (15.2%)	1.233.000 (7.4%)	2.140.000 (12.9%)
No beta-blockers	535 (84%)	15.130.000 (91.2%)	14.096.000 (85.0%)

a Numbers do not add up to 100% as not all beta-blockers could be specified. Source: Foundation for Pharmaceutical Statistics [17]. Source: Statistics Netherlands [18].

## Discussion

In a nationwide prospective genetic association study, we show an association of genetic variation in G protein-coupled receptors with susceptibility to bacterial meningitis [14]. The effect of *ADRB2* SNP rs1042714 was most clear for pneumococcal meningitis. We did not identify an association of rs1042714 with susceptibility to meningococcal meningitis and matched controls, although the difference in genotype frequencies between patients and controls was similar to that seen in pneumococcal meningitis. Further studies are needed to validate the identified associations in bacterial meningitis and pneumococcal meningitis, and determine the role of rs1042714 in a larger population of meningococcal meningitis patients. Furthermore, it would be interesting to compare meningitis patients with those that had bacteraemia due to the same pathogens, who did not develop meningitis.

The importance of G protein-coupled receptors for microorganisms to cross the blood-brain-barrier has been described *in vitro* in the binding of *S. pneumoniae* to the platelet activating factor receptor (PAFr), which facilitates transcytosis [8]. PAFr knockout mice showed to be protected against pneumococcal meningitis after intravenous injection of pneumococci [10]. Binding of *S. pneumoniae* to the β2-adrenoceptor has not been studied so far. The described molecular mechanisms of the interaction of *S. pneumoniae* and PAFr, and that of *N. meningitidis* and the β2-adrenoceptor are quite different, but in both processes β-arrestin-1 plays a crucial role [9]. The identified association between the *ADRB2* SNP and susceptibility to pneumococcal meningitis in our study suggests this receptor may be of similar importance for the pathophysiology of pneumococcal meningitis as was recently shown for meningococcal meningitis. As no interaction between the β2-adrenoceptor and the pneumococcus has been shown, our results must be interpreted with caution and regarded as explorative. It is likely that micro-organisms need multiple receptors to achieve sufficient adhesion to the endothelial cells and cross the blood-brain barrier [9]. Further experimental data are needed to determine the interaction of *S. pneumoniae* with the β2-adrenoceptor.

In this study we identified the β2-adrenoceptor as a potential target for therapy to prevent bacteria crossing the blood-brain barrier. The availability of the β2-adrenoceptor can be pharmacologically decreased by either binding of antagonists (beta-blockers) or downregulation of the receptor following catecholamine treatment [19]. It has been suggested that patients treated with catecholamines for meningococcal septic shock may benefit from downregulation of the receptor, as this will interfere with crossing of the blood brain barrier by the pathogen [19]. Prevention of bacterial invasion of the central nervous system (CNS) in sepsis patients is likely to reduce neurologic complications and improve outcome. The yield of this treatment strategy is probably limited, as concomitant initiation of antimicrobial treatment reduces the need for prevention of bacterial spread to the CNS. However, cases due to multiresistant bacteria may benefit from this approach.

Patients using non-selective beta-blockers were underrepresented in our patient cohort, while patients using selective beta-blockers were overrepresented, although this difference was not statistically significant. The additional blocking of β2-adrenoceptor by non-selective beta-blockers compared to selective beta-blockers may prevent bacteria crossing the blood-brain barrier and explain that patients on non-selective beta-blockers only developed meningitis when predisposing conditions such as otitis media or immunocompromised state were present. The association of beta-blockers with susceptibility to bacterial meningitis is however difficult to determine, since we have no data on the use of beta-blockers in the matched control population. Furthermore, an interaction between beta-blocker efficacy and rs1042714 has been reported that hinders a straightforward analysis [20]. The potential benefit of pharmacological treatment targeting the β2-adrenoceptor to prevent bacterial meningitis in patients with bacteraemia should be further studied in both experimental studies and observational case-control studies.

Our study has several limitations. First, the numbers of patients included in our study is relatively small for a study of polymorphisms. However, this is the largest genetic association study so far in bacterial meningitis patients, and provides interesting leads for further clinical and experimental studies. Furthermore, we did not replicate our findings in other case-control studies of adults with bacterial meningitis. Currently, no such studies are at our disposal to validate our findings. Third, we did not have information on the medical history of the control population and were therefore unable to correct for diseases that may result in confounding. Finally, in this study we show an association between rs1042714 and susceptibility but do functional demonstration this association is causal. Therefore, our results must be interpreted with caution.

In conclusion, we identified an association between rs1042714 *ADRB2* and susceptibility to bacterial meningitis. We have linked the β2-adrenoceptor/β-arrestin pathway with increased susceptibility to bacterial meningitis *in vivo*.
